# Recent Progress in Nanomaterials Modified Electrochemical Biosensors for the Detection of MicroRNA

**DOI:** 10.3390/mi12111409

**Published:** 2021-11-17

**Authors:** Sze Shin Low, Daizong Ji, Wai Siong Chai, Jingjing Liu, Kuan Shiong Khoo, Sadegh Salmanpour, Fatemeh Karimi, Balakrishnan Deepanraj, Pau Loke Show

**Affiliations:** 1 Research Centre of Life Science and Healthcare, China Beacons Institute, University of Nottingham Ningbo China, 199 Taikang East Road, Ningbo 315100, China; szeshin.low@gmail.com; 2 State Key Laboratory of Molecular Engineering of Polymers, Fudan University, Shanghai 200433, China; jidaizong@fudan.edu.cn; 3 School of Mechanical Engineering and Automation, Harbin Institute of Technology, Shenzhen 518055, China; cloudcws@gmail.com; 4 College of Automation Engineering, Northeast Electric Power University, Jilin 132012, China; 5 Faculty of Applied Sciences, UCSI University, UCSI Heights, Cheras 56000, Malaysia; kuanshiong.khoo@hotmail.com; 6 Department of Chemistry, Sari Branch, Islamic Azad University, Sari 1931848161, Iran; sadeghsalmanpour2017@gmail.com; 7 Department of Chemical Engineering, Quchan University of Technology, Quchan 9477177870, Iran; fkm024@gmail.com; 8 Department of Mechanical Engineering, Jyothi Engineering College, Thrissur 679531, India; babudeepan@gmail.com; 9 Department of Chemical and Environmental Engineering, Faculty of Science and Engineering, University of Nottingham Malaysia, Selangor 43500, Malaysia

**Keywords:** nanomaterial, electrochemical biosensor, MicroRNA, signal amplification strategy

## Abstract

MicroRNAs (miRNAs) are important non-coding, single-stranded RNAs possessing crucial regulating roles in human body. Therefore, miRNAs have received extensive attention from various disciplines as the aberrant expression of miRNAs are tightly related to different types of diseases. Furthermore, the exceptional stability of miRNAs has presented them as biomarker with high specificity and sensitivity. However, small size, high sequence similarity, low abundance of miRNAs impose difficulty in their detection. Hence, it is of utmost importance to develop accurate and sensitive method for miRNA biosensing. Electrochemical biosensors have been demonstrated as promising solution for miRNA detection as they are highly sensitive, facile, and low-cost with ease of miniaturization. The incorporation of nanomaterials to electrochemical biosensor offers excellent prospects for converting biological recognition events to electronic signal for the development of biosensing platform with desired sensing properties due to their unique properties. This review introduces the signal amplification strategies employed in miRNA electrochemical biosensor and presents the feasibility of different strategies. The recent advances in nanomaterial-based electrochemical biosensor for the detection of miRNA were also discussed and summarized based on different types of miRNAs, opening new approaches in biological analysis and early disease diagnosis. Lastly, the challenges and future prospects are discussed.

## 1. Introduction

MicroRNAs (miRNAs) are small size (19–24 nt) endogenous non-coding, single stranded RNAs which modulate the expression of their target genes. They possess high stability even in extreme conditions that cause degradation of most RNAs [1]. MiRNA was first discovered in *Caenorhabditis elegans* when the lin-4 gene expression was found to regulate the developmental stage of larvae, thereby revolutionizing the molecular biology field [2,3]. They play crucial regulatory roles in a wide variety of biological processes, such as cell differentiation, proliferation, control of developmental timing, organ development, apoptosis, and others [4]. MiRNA negatively regulate their targets by complementary binding to protein-coding messengerRNA sequences, activating the RNA-mediated interference (RNAi) pathway. On the other hand, miRNAs could also adhere to the 3′ untranslated regions (UTRs) of targets via imperfect complementary binding, repressing post-transcriptional gene expression, resulting in negative regulation [5]. 

For the past few years, miRNAs have been investigated in different types human cancers, where they are found to be heavily dysregulated in cancer cells, leading to cancer progression based on different mechanisms [6]. There has been report demonstrating that high-throughput profiling of genome-wide miRNA expression showed specific profile of upregulated and downregulated miRNAs for almost all cancer types [7]. Since deregulated miRNA expression is an early event in tumorigenesis, multiple studies have explored the potential usefulness of miRNA expression profiles as biomarkers for cancer diagnosis, prognosis, and response to treatment. Owing to the excellent stability, resistance to harsh environment, distinctive miRNA expression profile for every cancer, miRNAs possess huge potential of noninvasive biomarkers for early cancer detection, greatly reducing the worldwide health burden of cancer [8]. Therefore, the need for simple noninvasive detection of miRNA is essential for early detection of cancer, increasing the survival rate of cancer patients. 

Low abundance, short sequence length, high sequence homology, heterogeneity in GC contents and large number of isoforms of miRNAs present challenges in the designing of primer or probe for hybridization, making them very difficult for detection [9,10]. Traditional methods for miRNAs detection include Northern blotting, reverse transcriptase-polymerase chain reaction (RT-PCR), microarray technique and next-generation sequencing (NGS) and each method possesses unique advantages and disadvantages. For instance, Northern blotting is a widely used miRNA detection method that enables size detection and alternate splice product observation, but it is semi quantitative, less sensitive and low throughput. The gold standard miRNA detection method is RT-PCR due to its large dynamic range, high sensitivity, and specificity, it suffers from drawbacks such as false-positive anxiety and difficulty in primer design. On the other hand, a microarray-based technique displays high throughput and multiplexing capacity, but poor sensitivity and lengthy hybridization time limits its wide applications [11]. NGS is highly sensitive, high throughput and provides specific sequence information but it is very time-consuming and expensive [12]. In view of the pros and cons of different methods, the detection approach for miRNA continues to develop to meet the demands. 

The advancement in nanotechnology has led to their potential applications in various fields, including the development of biosensor. The nanomaterial-based electrochemical biosensors have become one of the spotlights in the field of diagnostics. With the growing demand for higher sensitivity and lower detection limit, nanomaterials could enhance the sensing performances due to their high surface area-to-volume ratio (SA:V), excellent electrical conductivity, and remarkable chemical stability [13,14]. Nanomaterials are generally used as working electrode modifier in biosensor, enabling sensitive detection of analyte as low as minute concentration [15]. Nanomaterials have been displayed to play an important role in the development of electrochemical biosensor for detection of various analytes, such as antibiotics, metabolic, cancer, virus, and miRNA biomarkers [16,17,18,19,20,21]. In this review, we provide an overview on the recent progress in the development of nanomaterial-based electrochemical biosensor for the detection of miRNA. The amplification strategies in miRNA biosensor were introduced, including cyclic enzymatic amplification method based on nuclease, rolling circle amplification (RCA) and enzyme-free amplification strategy. Nanomaterials have been employed for the modification of biosensing platform and electrochemically active labels for detection signal amplification. Recent works completed have been discussed based on different miRNA types and their sensing performances were summarized. Furthermore, the challenges and potential opportunities for the nanomaterial-based electrochemical miRNA biosensor are also proposed.

## 2. Amplification Strategies in miRNA Biosensor

Various amplification strategies have been employed in miRNA biosensor to achieve higher sensitivity and lower detection limit due to the generally low concentration of miRNA present in real sample. MiRNA biosensor typically relies on the hybridization between complementary probe and target miRNA sequence for detection. However, hybridization probes form a 1:1 complex with targets to produce signal, leading to low signal being generated, which is insufficient for the sensitive detection of low abundance miRNA. Therefore, various signal amplification strategies have been developed to replicate the target miRNA by 10^8^–10^9^-fold, achieving increased signal generation, thereby improving the detection limit of miRNA [22,23].

### 2.1. Cyclic Enzymatic Amplification Method (CEAM) Based on Nuclease

Cyclic enzymatic amplification method (CEAM) based on nuclease is frequently employed for the sensitive detection of nucleic acids, where nuclease cleaves the duplex nucleic acid, releasing the one target for recycling, thereby achieving output signal amplification. CEAM is a simple, rapid and inexpensive method often employed in biosensing platform design for highly sensitive detection of miRNA as reported in review [24].

**Figure 1:**
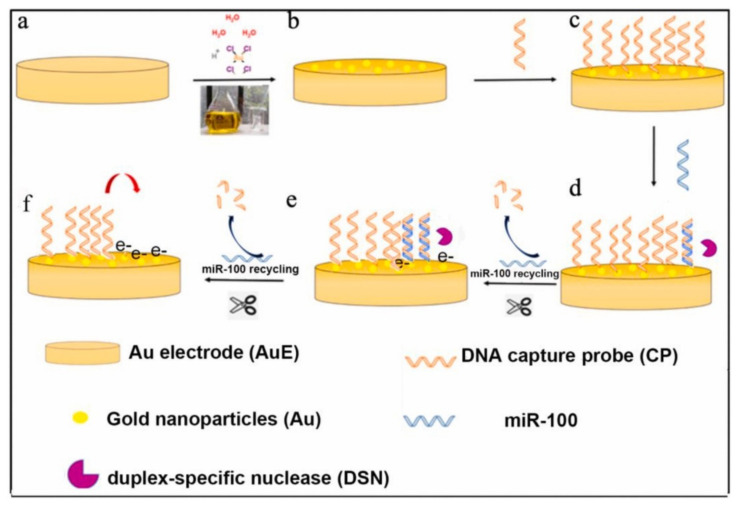
Schematic workflow for electrochemical detection of miR-100 using AuNPs-modified AuE coupled with DSN-assisted target recycling for signal amplification. Reprinted from [28] with permission from Elsevier.

Duplex specific nuclease (DSN) is an enzyme that was originally isolated from hepatopancreas of the Kamchatka crab (*Paralithodes camtschaticus*) and was applied for single nucleotide polymorphism (SNP) detection [25]. It is typically used in the normalization of the relative transcript abundance in mRNA-enriched cDNA libraries from eukaryotic organisms, quantitative determination of telomeric overhang [26,27]. DSN preferentially cleaves double-stranded DNA (dsDNA) or DNA in the DNA:RNA heteroduplexes at high temperatures while inactive towards single-stranded DNA and single- or double-stranded RNA. The good ability of DSN in discriminating between perfect and imperfect (up to one base mismatch) matched short duplexes has rendered its usefulness in biosensor. Recent work displayed that a gold nanoparticle (AuNP)-modified gold electrode was coupled with DSN-assisted target recycling for sensitive detection of miR-100 in the linear range of 100 aM to 10 pM as displayed in Figure 1 [28]. Results showed that the biosensor was highly specific, illustrating the ability to distinguish one base-pair mistake in miR-100 detection. Furthermore, the 5 fM cutoff miR-100 concentration in human sera was sufficient for detection of gastric cancer in clinical applications.

Exonuclease III (Exo III) is an enzyme isolated from *Escherichia coli* that possesses phosphomonoesterase activity specific for 3′ positions of double-stranded nucleic acid [29]. It catalyzes the degradation of double-stranded DNA terminating in 3′-OH, performing stepwise removal of mononucleotides from DNA in the 3′ to 5′ direction. Exo III degrades dsDNA from blunt ends, 5′-overhangs or nicks and produces stretches of single-stranded DNA. Noteworthily, Exo III can digests dsDNA from the 3′ end even though it contains up to three base mismatched but fails to initiate digestion for dsDNA terminating in four base protrusions [30]. On the other hand, T7 exonuclease is also widely applied in CEAM as it catalyzes the removal of mononucleotide from the 5′-phosphorylated and 5′-hydroxylated end to the 3′ end of dsDNA [31]. Contrary to Exo III, T7 exonuclease hydrolyzes dsDNA in the 5′ to 3′ direction but it is unable to act on dsDNA with protruding ends. Both Exo III and T7 nuclease have been applied in biosensor for the sensitive detection of miRNA based on CEAM [32,33,34,35].

### 2.2. Rolling Circle Amplification (RCA)

RCA amplification is an isothermal enzymatic process for the amplification of circular nucleic acid [36]. RCA utilizes DNA/RNA polymerase with strand displacement activity to extend short DNA or RNA primer annealed to the circular nucleic acid template. The polymerase performs strand displacement activity to release single-stranded DNA for further amplification, resulting in the generation of long single-stranded oligonucleotide with repeated sequence complementary to the circular template. The end product is a concatemer containing tens to hundreds of tandem repeats, presenting RCA as a highly versatile amplification tool with wide range of applications in genomics, proteomics, biosensing, and more [37,38]. RCA is also applied in miRNA detection where Jonstrup et al. (2006) developed a padlock exponential rolling circle amplification using the target miRNA as template, performed the detection of specific miRNA in a few nanograms of total RNA without specialized equipment and a detection limit of 10 pM was achieved [39]. Although RCA is versatile and capable of producing tailor-designed nucleic acids or complex nanostructures, its limited sensitivity and time-consuming process still requires improvement to satisfy the detection need.

### 2.3. Enzyme-Free Amplification Strategy

**Figure 2:**
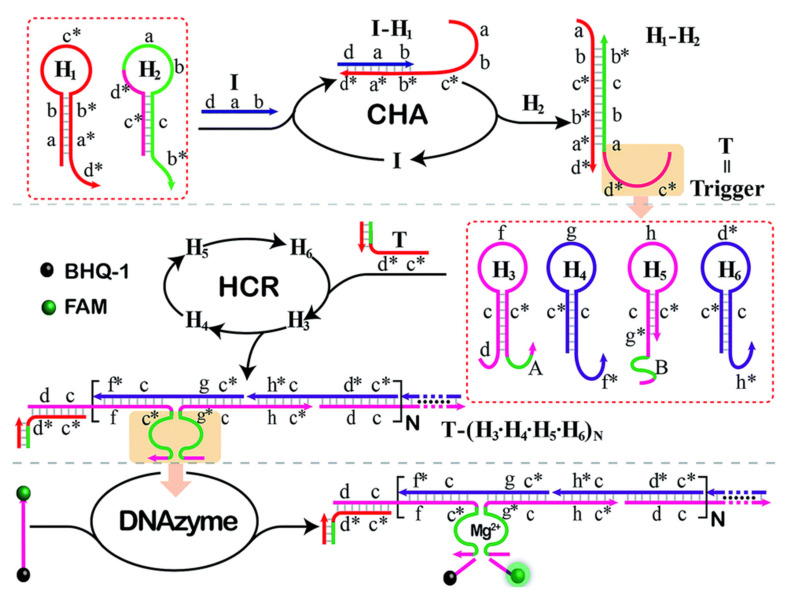
Schematic diagram showing an example of cascade amplification strategy involving catalyzed hairpin assembly (CHA) and hybridization chain reaction (HCR) where both strategies also employ hairpin probes for signal amplification. Reprinted from [46] with permission from Royal Society of Chemistry.

Hybridization chain reaction (HCR) displays great potential in biosensor signal amplification as it provides an attractive approach to enzyme-free nucleotide amplification [40]. It is a kinetics-controlled reaction with high sensitivity and selectivity for target sequence detection. HCR triggers the DNA nanostructures self-assembly based on the potential energy in two hairpin species [41]. In the presence of target nucleic acid, one hairpin species will be opened up and expose the region that could trigger the opening of another hairpin species, as shown in Figure 2. The process kept on repeating, leading to the formation of nicked double helix that grows until hairpin supply is exhausted. HCR is often employed for the detection of miRNA as it can trigger a series of cascade hybridization chain reaction, leading to extended growth of oligonucleotide chains and higher amplification efficiency [42,43,44]. Generally, HCR is an effective signal amplification strategy as it is enzyme-free and can bind thousands of auxiliary hairpin sequences. However, it suffers from drawbacks, such as laborious labeling techniques or strict requirement for reaction environment [24].

Catalyzed hairpin assembly (CHA) is also an enzyme-free signal amplification strategy that is based upon DNA nanostructure organization. It is a highly efficient isothermal amplification method that is widely utilized in amplifying and transducing signals for nucleic acid analyte towards biosensing [45]. CHA is similar to HCR where the presence of single-stranded target analyte initiates the opening of hairpins, resulting in thermodynamically stable duplexes. CHA is often used with HCR to achieve desired signal amplification as illustrated in Figure 2 [46]. Studies have proved that CHA could achieve up to 600,000-fold signal amplification under certain conditions, displaying its promising versatility [47]. Nevertheless, it is difficult to completely prevent undesired hybridization event in the absence of an input, culminating the high background noise, which might compromise the performance of CHA. 

### 2.4. Intercalation of Redox Mediator

Redox mediators play pivotal roles in enhancing the performance of electrochemical sensors due to the electron transfer process involved in the redox reactions. In electrochemical biosensors, redox mediators are often intercalated between two oligonucleotide strands as signal indicators. The redox mediators could be intercalated via direct and indirect method, where the former relies on the interaction of electroactive species with double-stranded oligonucleotide and the latter involves the creation of recognition sites in oligonucleotide for intercalation [48]. Methylene blue (MB) is a popular electroactive indicator used as DNA intercalating agent as it could interacts with both single-stranded and double-stranded oligonucleotide with different binding modes. Therefore, MB is often employed for sensitive electrochemical detection of microRNA due to its signal amplification properties. For instance, an electrochemical sensor modified using graphene, polypyrrole, and gold nanoparticles was applied for the detection of miR-21 based on intercalating MB signal amplification [49]. The peak current of MB redox process was proportional to miR-21 concentration, achieving low detection limit of 0.02 fM. On the other hand, a newly discovered G-triplex that can specifically bind with MB was applied in electrochemical detection of let-7a by coupling with two-stage isothermal exponential amplification reaction, displaying ultralow detection limit of 0.45 fM and high specificity [50]. 

## 3. Nanomaterial-Based Electrochemical Biosensors for miRNA Detection

Electrochemical biosensors have been developed for detection of various miRNA biomarkers due to the ease of electrochemical detection, low cost, and potential for multiplexed platforms. The emerging field of nanomaterial-based electrochemical biosensors has been gaining attention among the scientific community due to the outstanding electrochemical properties of nanomaterials, along with good biocompatibility, chemical stability, and large SA:V. Most of the electrochemical biosensors rely on complementary hybridization between oligonucleotide strands for miRNA detection, on top of signal improvement via nanomaterial modifications. The introduction of nanomaterials into electrochemical biosensors could enhance the precision and accuracy for miRNA detection, serving as the electrode materials in biosensor for enhanced sensitivity, generation of active sites for immobilization of biological molecules, signal amplifier in a series of hybridization events, catalyst for electrochemical reaction, electroactive labels [19]. The most commonly detected miRNA biomarkers are miR-141, miR-21, miR-155, and more. 

### 3.1. Detection of miR-21

**Figure 3:**
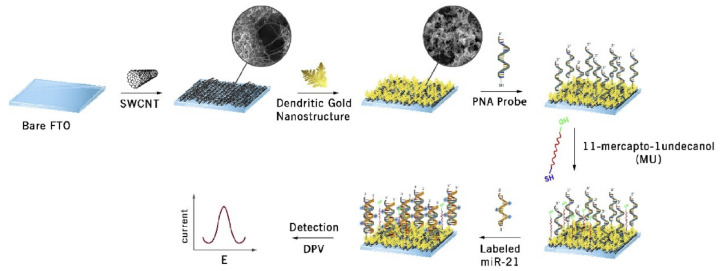
Schematic presentation of the various steps involved in biosensor modification with single-walled carbon nanotubes (SWCNTs) and dendritic gold nanostructure for the electrochemical sensing of miR-21 via differential pulse voltammetry (DPV). Reprinted from [52] with permission from Elsevier.

Different types of miRNAs have been identified and their sequence information could be obtained from various databases, including miRBase, miRVIT, miRDB, and more. Among numerous miRNAs types, miR-21 has been studied extensively and identified to be overexpressed in various types of cancerous tumors, including stomach, prostate, head and neck, pancreas, lung, colorectal, esophagus, glioblastoma, and neuroblastoma [51]. Gold nanoparticles (AuNPs) are often employed in biosensing platform due to the excellent catalytic and conductive properties. Sabahi et al. (2020) modified bare fluorine doped tin oxide (FTO) electrode with single-walled carbon nanotube (SWCNT) and dendritic gold nanostructure for the detection of miR-21 with Cadmium ions (Cd^2+^) as signal units as illustrated in Figure 3 [52]. Low detection limit (0.01 fM) was achieved and the fabricated biosensor also displayed acceptable performance in human serum samples. Gold nanoparticles functionalized with MoS_2_ nanosheet (NS) were synthesized using thionine as reducing agent for label-free detection of miR-21 in the linear range of 1 pM to 10 nM and detection limit of 0.26 pM [53]. Reduced graphene oxide/gold nanoparticles (rGO/AuNPs) were also employed for the detection of spiked miR-21 in artificial saliva with good recovery rate ranging from 96.2% to 107.2% [54]. In another study, virus-like hallow structure of CuCo_2_O_4_ was applied to modify the screen carbon printed electron-gold nanoparticle (SCPE-AuNP) electrode surface to hold large amounts of p19 protein, mimicking the inherent virus (Carnation Italian ringspot virus) for the detection of miR-21 [55]. The fabricated biosensor displayed good sensing performance when applied in human serum, MCF-7, and HeLa cells with high sensitivity, specificity and reproducibility in few minutes. The same research group also tested the SCPE-AuNP electrode for miR-21 detection from MCF-7 exosome based on the multi-covalent attachment of p19 protein [56]. It was the first time that the exosomal electrochemical properties were proven as the electrochemical amplifier bed, achieving detection limit of 1 aM. Apart from direct modification of electrode surface with nanomaterials, multi-walled carbon nanotubes (MWCNTs) subjected to different treatments were applied in miR-21 biosensor [57]. The loading capacity of thionin on the MWCNTs was investigated to determine the good electrochemical signal nanoprobes, resulting in low detection limit of 0.032 pM.

**Figure 4:**
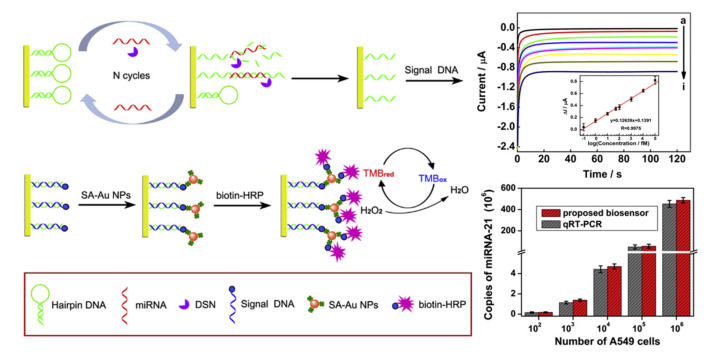
Electrochemical biosensor for miR-21 detection based on triple signal amplification approach, displaying excellent sensitivity with sensing performance comparable to that of qRT-PCR. Reprinted from [60] with permission from Elsevier.

Owing to the low concentration of miRNA in sample, most of the electrochemical biosensing design incorporate amplification strategy in the detection mechanism. Amplification strategy that involves the recycling of target is highly sensitive and specific as the target miRNA is essential for the initiation of amplification cycle. For instance, Chen et al. (2019) performed T7 exonuclease-assisted target recycling for ultrasensitive electrochemical detection of miR-21 based on the one-step biorecognition reaction at vertically aligned SWCNTs [58]. In the presence of target miR-21, the ferrocene-labeled DNA probe hybridized with miR-21 to form duplex that was later release from the electrode surface, leading to corresponding decrease in electrochemical signal. T7 exonuclease cleaved the duplex and recycled the target miR-21 for further hybridization, amplifying the signal to a low detection limit of 3.5 fM. On the other hand, DSN-assisted target recycling is employed for ultrasensitive electrochemical detection of miR-21 using multi-walled carbon nanotubes@graphene oxide nanoribbons/gold nanoparticles (MWCNTs@GONRs/AuNPs)-modified biosensor [59]. Dual amplification strategy was carried out by the addition of streptavidin-conjugated alkaline phosphatase (SA-ALP)-immobilized DNA probe, showing satisfactory sensitivity (detection limit, 0.034 fM) and good accuracy (recovery ratio, 77.4–120.2%). Zhang et al. (2019) further enhance the sensitivity by performing triple signal amplification approach, including DSN-assisted target recycling followed with AuNPs and horse radish peroxidase (HRP) amplification [60]. As shown in Figure 4, the presence of target miR-21 opened the hairpin DNA and formed duplex which was recognized and cleaved by DSN. The residual DNA fragment on electrode hybridized with signal DNA and attracted the binding of AuNPs through streptavidin–biotin interaction. The Au NPs served as nanocarriers for HRP and maintained their enzymatic activity, where the HRP catalyzed the reduction of hydrogen peroxide to generate electrochemical current signal, presenting a low detection limit of 43.3 aM.

**Figure 5:**
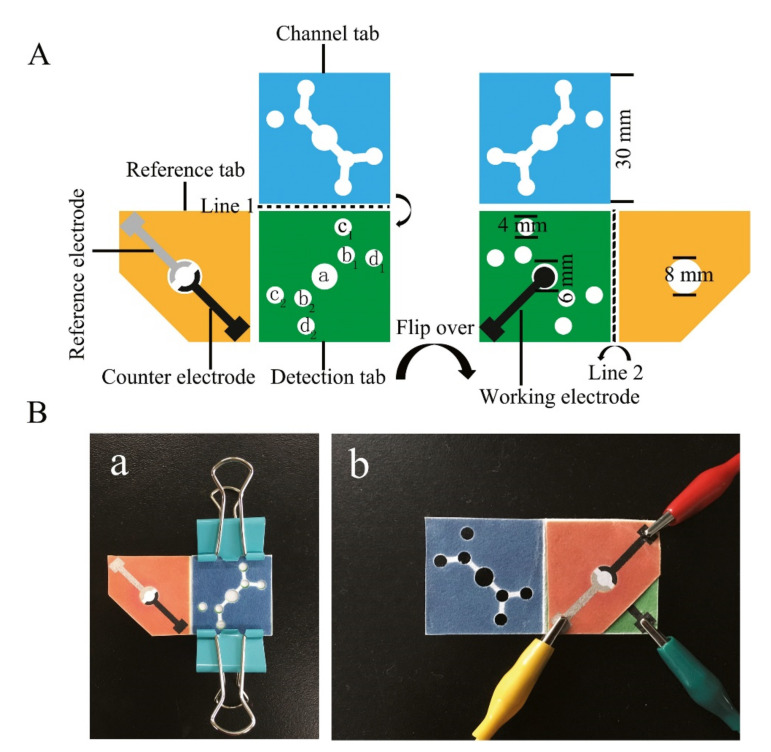
(**A**) Schematic representation and (**B**) fabrication of μPADs for the detection of miRNA combining chromogenic reaction and electrochemistry. Reprinted from [66] with permission from Elsevier.

Padlock exponential rolling circle amplification (P-ERCA) combined with CoFe_2_O_4_ magnetic nanoparticles (MNPs)-assisted non-substrate nanoelectrocatalysis were applied in the biosensing platform design for the detection of miR-21 [61]. In order to improve the catalytic efficiency, CoFe_2_O_4_ MNPs and redox molecule (toluidine blue) were co-immobilized onto graphene surface, significantly improving the detection sensitivity with a wide dynamic range of 1 fM to 2 nM. Furthermore, CHA is also employed in conjunction with Pd-based nanomaterials (Pd NPs, Pd@UiO-66) for the ultrasensitive detection of miR-21, providing a powerful sensing platform [62,63]. Noteworthily, the utilization of metal-organic framework (MOF) UiO-66 is attractive as the combination of inorganic metal ions and organic linkers have demonstrated extraordinary performance due to well-defined porosities, high thermal stability, large surface area, and chemical tunability. Hu et al. (2018) also performed target-recycled non-enzymatic amplification for in vitro miR-21 sensing in a broad linear dynamic range of 0.1 fmol to 5 pmol and a detection limit of 56.7 amol [64]. Interestingly, paper-based electrochemical biosensor was develop to present a rapid, portable and renewable method for convenient and sensitive detection [65]. A microfluidic paper-based analytical device (μPAD) shown in Figure 5 was constructed using cerium dioxide–Au@glucose oxidase (CeO_2_-Au@GOx) acting as the signal transducer layer [66]. The μPADs provided a wide linear range of 1 to 1000 fM with a relatively low detection limit of 0.434 fM. Zhao et al. (2021) utilized guanine-quadruplex (G-quadruplex) formation in nanochannels for label-free electrochemical biosensing of miR-21 [67]. Carbon nanofibers-modified electrode was applied to monitor the change in electrochemical signal generated from the methylene blue fluxed through the nanochannels due to the presence of miR-21. A novel relay-race mechanism based on RNA/barcode gold nanoflower hybrid was proposed to enhance the electrochemical signal by ~230 times [68]. The fabricated sensor only requires small sample volume of 4 μL and could operate in a wide linear range of 500 aM to 1 μM, successfully profiling two cancer serums (breast and liver) at different development stages based on the miR21 abundance differences.

### 3.2. Detection of miR-144 and miR-200a

**Figure 6:**
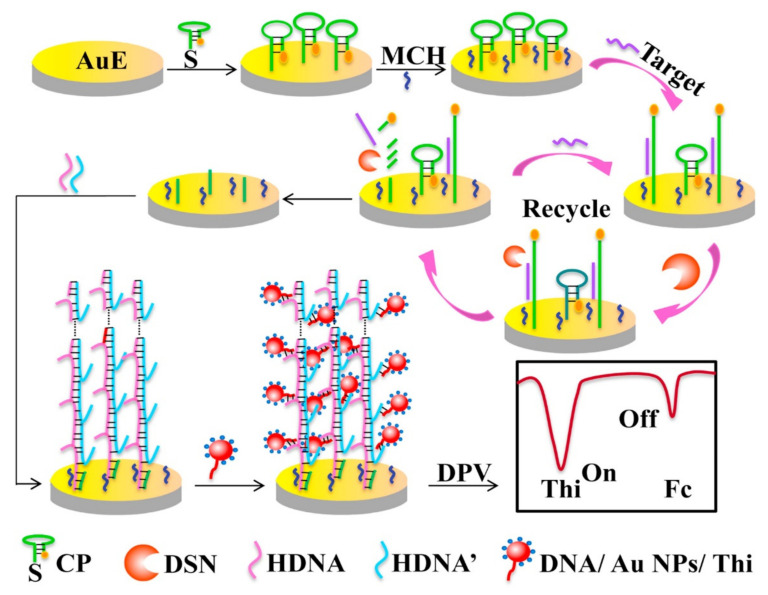
Schematic diagram illustrating the ratiometric electrochemical assay for miR-141 detection based on DSN and HCR amplification. Reprinted from [72] with permission from Elsevier.

MiR-144 and miR-200a belong to the miR-200 family, which is associated with the formation of cancer steam cells and regulation of epithelial-mesenchymal transition (EMT), increasing the cancer cell motility and invasiveness [69]. MiR-141 is commonly dysregulated in malignant tumors and the detection of its concentration is important in monitoring tumor development and progression. Miao et al. (2018) employed T7 exonuclease-assisted cascade signal amplification and DNA-templated copper nanoparticles (CuNPs) for the electrochemical detection of miR-141 [34]. The nucleic acid duplex served as template for the in situ synthesis of CuNPs as excellent electrochemical signal sources, the addition of miR-141 triggered T7 exonuclease activity and decreased the electrochemical signal, displaying good detection linear range of 10^−16^ to 10^−13^ M and detection limit of 4.5 × 10^−17^ M. The same research group further developed the electrochemical miRNA biosensor based on DNA-functionalized porous Fe_3_O_4_ nanoparticles with target recycling amplification [70]. Redox probe [Fe(CN)_6_]^3−^ entrapped inside porous Fe_3_O_4_ nanoparticles by DNA probe was released upon hybridization with target miR-141, significantly increasing the open circuit voltage, performing “signal-on” self-powered biosensor with good detection limit (1.4 aM), demonstrating great potential as a powerful tool for miRNA diagnostics. Ratiometric biosensor displays greater anti-interference ability and measurement accuracy, which has also been employed for miRNA detection [71]. Yuan et al. (2018) designed a ratiometric electrochemical miR-141 assay based on dual-amplification mechanism (DSN, HCR) as shown in Figure 6 [72]. The ratiometric signal was obtained from the electrochemical signal of thionine and ferrocene, providing a detection limit down to 11 aM. This dual-amplified ratiometric biosensor possesses intrinsic self-calibration ability to remove the fluctuations from the system, which is promising for diagnostic applications. Another member of miR-200 family, miR-200a is a potential ovarian cancer biomarker and thus femtomolar determination of miR-200a in blood plasma using label-free electrochemical biosensor based on L-cysteine functionalized ZnS quantum dots has been performed [73]. The functionalized nanomaterial acted as suitable substrate for immobilization of DNA probe and enhance the sensing performance to a wide linear detection range of 1 × 10^−14^ to 1 × 10^−6^ M.

### 3.3. Detection of Other miRNAs

Research works have been reported on the detection of various miRNAs as they are the potential biomarkers for different types of diseases. Most of the works employed nuclease-assisted signal amplification strategy for the improved sensing performance to detect low concentration of miRNA. The incorporation of nanomaterials in addition to signal amplification strategy significantly enhanced the sensitivity and detection limit of electrochemical miRNA biosensor, such as the modification of electrode surface with AuNPs/Ti_3_C_2_ MXene, AuNPs, addition of Ag-PEI NPs as electroactive label and addition of nanoscale copper based metal organic framework assembled Pt NPs and horseradish peroxidase (Cu-NMOF@PtNPs/HRP) as catalytic nanoprobe for the detection of miR-155 [32,74,75,76]. Similarly, AuNPs, cysteamine-capped AuNPs, amino-functionalized graphene quantum dots (GQDs), graphene oxide (GO), electrochemically-reduced graphene oxide/gold nanowires (ERGO/AuNRs), GO/AuNRs, black phosphorus nanosheets/thionine-doped copper-MOF (BPNSs/TH/Cu-MOF) and C_60_@PAMAM-MOF were applied to modify the electrode surface for sensitive detection of miR-103, miR-25, miR-34a, miR-137, miR-199a-5p, miR-3123, and miR-3675-3p [77,78,79,80,81,82,83,84,85]. Furthermore, nanomaterials also serve as electroactive label for enhancing the electrochemical signal generation. Silver nanoparticles/single-walled carbon nanotubes (AgNPs/SWCNTs) nanohybrid and doxorubicin-loaded AuNPs were introduced as label to that could interact with the duplex nucleic acid for sensitive detection of miR-25 in human plasma and let-7d [86,87]. Both biosensors displayed good sensing performances with detection limit of 3.13 × 10^−13^ M and 0.17 pM for miR-25 and let-7d, respectively.

### 3.4. Simultaneous Detection of miRNAs

**Figure 7:**
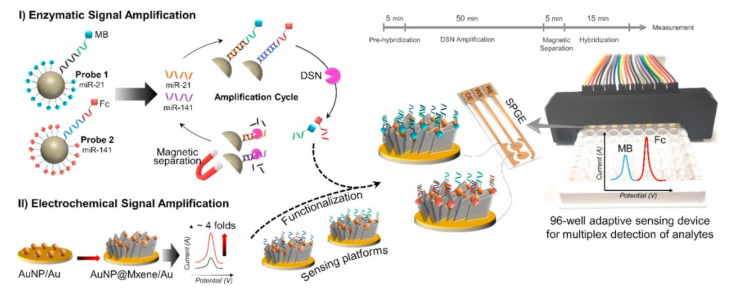
Schematic diagram representing the biosensing assay for simultaneous detection of miR-21 and miR-141. (**I**) Enzymatic signal amplification was performed based on DSN-assisted target recycling approach followed by magnetic separation; (**II**) Electrochemical signal amplification was achieved via the Au electrode modification with AuNP@MXene. Reprinted from [89] with permission from Elsevier.

Simultaneous multiplex miRNA biomarker detection is in great demand for early and accurate cancer diagnosis. MiR-21 and miR-141 are often detected simultaneously using different reporter molecules, or different types of nanomaterials, which could generate different electrochemical signal output for multiplex detection. Tian et al. (2019) constructed MoS2/AuNPs/AgNW paper-based electrode as biosensor for simultaneous detection of miR-21 and miR-141 using ferrocene and methylene blue as the reporter molecule [88]. Hierarchical assembled nanomaterials and MOF (PtCuMOFs) were further immobilized on the DNA probe for signal enhancement as the combination of multi-dimensional nanomaterials contributes to low impedance and large sensing area. Under optimal conditions, simultaneous detection of miR-21 and miR-141 with detection limit of 0.1 fM was demonstrated. Similar work was reported for the simultaneous detection of miR-21 and miR-141 towards point of care cancer screening as shown in Figure 7, where AuNP@Mxene was employed for the modification of biosensor [89]. DSN-based amplification assay was applied, enhancing the sensing sensitivity (detection limit: 204 aM for miR-21; 138 aM for miR-141) and single-mutation recognition ability. The DNA probe was labeled with ferrocene and methylene blue which generate distinguishable electrochemical signal, providing multiplexability for the biosensor.

On the other hand, Yuan et al. (2017) paired up ferrocene with thionine as the reporter molecule for simultaneous detection of miR-21 and miR-141 [90]. Target-triggered HCR strategy was introduced to improve the sensitivity, enabling the sensor to detect miRNAs in cell lysate. In another work, MOF UIO-66-NH_2_ was applied as nanocontainer for the loading of electroactive dyes (methylene blue and TMB), which was used in the simultaneous detection of let-7a and miR-21 [91]. A specially designed oligonucleotide that is complementary to target miRNA acted as the gatekeeper to form dsDNA-capped MOFs, which released the electroactive dyes upon the presence of miRNA triggering the toehold-mediated strand displacement reaction. Thus, detection limits of 3.6 and 8.2 fM have been demonstrated for let-7a and miR-21, respectively, which are comparable or even lower than other reported strategies. Apart from the reporter molecule, nanomaterials were also employed to generate differential signal that is suitable for multiplex detection of miRNA. Electrochemically encoded responsive nanolabels comprising biotinylated molecular beacons and AuNPs or AgNPs were applied to generic neutravidin biosensor for the simultaneous multiplexed detection of miR-21 and miR-141 [92]. Stripping square-wave voltammetry (SSWV) was performed to analyze the electrochemical signal generated from the gold and silver label, resulting in limit of detection of 0.3 and 10 pM for miR-21 and miR-141, respectively. On an interesting note, the development of biosensor towards portable, miniaturized system could be assisted with self-powered system. Wang et al. (2018) fabricated a Nitrogen-doped hollow carbon nanospheres-based high-energy-density biofuel cells for self-powering simultaneous detection of miR-21 and miR-141, displaying detection limits as low as 0.1 and 4.0 fM, respectively [93]. This work has excellent potential in the development of economical and portable self-powered biomedical sensors.

Table 1 summarizes the performances of nanomaterial-based electrochemical biosensors for various miRNA targets detection.

## 4. Conclusions and Future Perspectives

The advancement of nanotechnology has contributed positively to the progression of biosensor development and fabrication. Significant progress has been made towards the development of advanced nanomaterial-based electrochemical biosensor for the detection of miRNAs over the last decades. Nanomaterials with unique properties, such as SA:V, good chemical stability, conductivity, electrocatalytic activity displayed promising results in enhancing the sensitivity and lowering the detection limit of target miRNAs through synergistic effects. The applications of signal amplification strategies based on enzymes and oligonucleotides have also successfully improved the detection sensitivity and specificity. The mechanisms of different signal amplification strategies were discussed to provide understanding on the key concept. The combination of signal amplification strategies and nanomaterial modifications have significantly enhanced the sensing performance that could be applied for detection of miRNA in complex matrix or clinical sample. However, there are still many challenges in the development of nanomaterial-based electrochemical miRNA biosensor and future research direction should gear towards the solution for these problems.

(i) Coupling of novel nanomaterials with recognition element—The progress in nanomaterial synthesis will lead to the construction of novel nanomaterials with desired properties that are suitable for application in electrochemical biosensor. Therefore, the coupling of novel nanomaterials with recognition element for miRNA biosensor, which is normally complementary DNA probe will lead to the fabrication of biosensor with high sensitivity and low detection limit. In addition, novel nanomaterials could also be applied as nanolabels that bind specifically to duplexes, generating enhanced electrochemical signal output and multiplex detection capability.
(ii) Reproducibility of nanomaterial-based electrochemical biosensor—The modification of biosensor with nanomaterials could improve the sensing performance of the biosensor, but there might be variation among each biosensor that was modified with the same nanomaterial. This could be due to the variation in the conformation or topology of modified nanomaterial on the biosensor surface, which is associated with the increased complexity of modified surface, giving rise to the reproducibility issue. Therefore, it is possible to perform statistical sampling on a batch of fabricated sensor and apply the testing and calibration to the entire batch.
(iii) Validation of miRNA biosensor via real sample detection—It is crucial that the miRNA biosensor could function in clinical sample, providing accurate and reliable results as diagnostic tool. As miRNAs are present in various bodily fluids, for example saliva, plasma, tear, interstitial fluid, serum, urine, and others, the clinical samples obtained will be complex matrices, which will interfere the detection and recovery ratio. In addition, real sample often contains many species that might affect the electrochemical process or non-specific adsorption to sensing surface. Therefore, innovative materials and methods need to be developed to create boundary or functional linkage for the specific adhesion of target analyte to the sensor surface, ensuring the accuracy and recovery of biosensor.

The ideal electrochemical biosensor for miRNA detection should be facile, efficient, highly sensitive and specific towards target miRNA, providing simple and rapid sensing for diagnostic purpose. Various signal amplification strategies could be employed to achieve the ideal goal, overcoming false positive and providing accurate detection. The incorporation of nanomaterials also contributed in enhancing the sensing performance due to their desired properties. It is anticipated that the future research direction could aid in solving the problems associated with nanomaterial-based electrochemical miRNA biosensor, providing a new perspective for miRNA sensitive detection, which is critical in biomedical applications development. 

## Figures and Tables

**Table 1:** Sensing performances of nanomaterial-modified electrochemical biosensor for the detection of miRNAs.

miRNA	Nanomaterial Used	Linear Range	Limit of Detection (LOD)	Electrochemical Technique	Remark	Ref
miR-21	Pd@UiO-66	20 fM to 600 pM	0.713 fM	DPV	CHA amplification	[63]
	SWCNT	0.01 to 100 pM	3.5 fM	DPV	T7 Exonuclease-Assisted Cascade Signal Amplification	[58]
	AuNPs	0.1 fM to 100 pM	43.3 aM	Amperometry	Triple amplification via DSN-assisted target recycling combined with gold nanoparticles, and horseradish peroxidase (HRP) enzymatic catalysis	[60]
	CNTs	1 fM to 1 μM	-	DPV	Target-induced synthesis of Mg2+-dependent DNAzyme	[65]
	GO; Pd NPs	1 fM to 50 pM	63.1 aM	DPV	CHA amplification	[62]
	AuNPs; CoFe_2_O_4_ MNPs	1 fM to 2 nM	0.3 fM	SWV	Padlock exponential rolling circle amplification (P-ERCA)	[61]
	GNF@Pt	1 μM to 500 aM	135 aM	DPV	Relay-race RNA/barcode gold nanoflower hybrid	[68]
	AuNPs	1 aM to 500 pM	1 aM	DPV	Exosomal electrochemical properties as electrochemical amplifier bed	[56]
	AuNRs; CeO_2_-Au@GOx	1 fM to 1000 fM	0.434 fM	DPV	-	[66]
	SWCNTs/dendritic Au	0.01 fM to 1 μM	0.01 fM	DPV	-	[52]
	MWCNTs-COOH	0.1 fmol to 5 pmol	56.7 amol	DPV	Target-recycled non-enzymatic amplification	[64]
	MoS2-Thionine-AuNPs	1.0 pM to 10.0 nM	0.26 pM	SWV	-	[53]
	rGO/Au	0.1 mM to 1 pM	1 pM	DPV	Smartphone-based portable electrochemical biosensing system	[54]
	Carbon nanofibers	1 aM to 10 pM	0.5 aM	DPV	Label-free sensing based on guanine-quadruplex (G-quadruplex) formation	[67]
	AuNPs; MWCNTs	0.1 to 12000 pM	0.032 pM	DPV	-	[57]
	CuCo_2_O_4_	100 fM to1 aM	1 aM	DPV	Virus-like hollow structure of CuCo2O4 filled with p19 protein	[55]
	MWCNTs@GONRs/AuNPs	0.1 nM to 0.1 fM	0.034 fM	DPV	DSN amplification	[59]
miR-141	AuNPs	0.1 fM to 100 pM	11 aM	DPV	Dual-amplification: DSN, HCR	[72]
	CuNPs	0.1 pM to 0.1 fM	0.45 aM	DPV	T7 Exonuclease-Assisted Cascade Signal Amplification	[34]
	GO/AuNPs/Gox; Fe_3_O_4_ NPs	10 aM to 10 fM	1.4 aM	EIS	Self-powered system with DSN amplification	[70]
miR-155	AuNPs	-	3.57 fM	SWV	Dual amplification via DSN amplification and strand displacement reaction	[74]
	Ag-PEI NPs	2 × 10^−20^ to 2 × 10^−12^ mol	20 zmol	CV	-	[75]
	Cu-NMOF@PtNPs	0.50 to 1.0 × 10^5^ fM	0.13 fM	SWV	Synergistically catalytic nanoprobe coupled with improved cascade strand displacement reaction	[76]
	AuNPs/Ti_3_C_2_ Mxene	10 nM to 1 fM	0.35 fM	DPV	Exonuclease III-aided cascade target recycling	[32]
miR-103	AuNPs	100 fM to 5 nM	100 fM	SWV	Label-free and reagentless detection	[77]
miR-25	Cysteamine-AuNPs	1 pM to 0.1 nM; 0.1 nM to 1 µM	0.25 pM	EIS	-	[78]
	AgNPs/SWCNTs	1 pM to 0.1 nM; 0.1 nM to 0.1 10 nM	0.313 pM	DPV	-	[86]
	Amino-functionalized GQDs	0.3 nM to 1.0 μM	95.0 pM	DPV	Accumulation of *p*-Biphenol	[79]
miR-34a	GO	5 to 35 μg/mL	7.52 μg/mL	DPV	-	[80]
	GO	0 to 10 µg/mL	261.7 nM	EIS	-	[81]
miR-137	ERGO + AuNWs	5 to 750 fM	1.7 fM	DPV	-	[82]
miR-200a	L-cysteine functionalized ZnS QDs	1 µM to 10 fM	8.4 fM	EIS	-	[73]
miR-199a-5p	GO-AuNRs	15 fM to 148 pM	4.5 fM	EIS	-	[83]
miR-3123	BPNSs/Thionine/Cu-MOF	2 pM to 2 μM	0.3 pM	SWV	-	[84]
miR-3675-3p	C_60_@PAMAM-MOF; Au@PtNPs	10 fM to 10 nM	2.99 fM	DPV	-	[85]
Let-7d	AuNPs@Doxorucibin	1 pM to 10 nM	0.17 pM	SWV	Double-loop hairpin probe	[87]
Simultaneous detection: miR-141 and miR-21	Fe_3_O_4_ NPs	1 nM to 1 fM	0.44 fM (miR-141) 0.46 fM (miR-21)	DPV	HCR amplification	[90]
	MoS_2_/AuNPs/AgNW	1 nM to 1 fM	0.1 fM	SWV	-	[88]
	AuNPs; AgNPs	50 to 1000 pM (miR-141); 0.5 to 1000 pM (miR-21)	10 pM (miR-141); 0.3 pM (miR-21)	SSWV	Neutravidin—biotin affinity	[92]
	AuNPs/Mxene	500 aM to 50 nM	138 aM (miR-141); 204 aM (miR-21)	DPV	DSN amplification	[89]
Simultaneous detection: miR-21 and let-7a	pNHCSs	0.1 nM to 3.16 fM	4.0 fM (miR-141); 0.1 fM (miR-21)	EIS	High-energy-density biofuel cells for self-powered sensing	[93]
	UIO-66-NH_2_	0.01 to 100 pM	8.2 fM (miR-21); 3.6 fM (let-7a)	DPV	-	[91]

AuNPs—gold nanoparticles; DPV—differential pulse voltammetry; DSN—duplex-specific nuclease; HCR—hybridization chain reaction; CuNPs—copper nanoparticles; GO—graphene oxide; Gox—glucose oxidase; EIS—electrochemical impedance spectroscopy; CHA—target-catalytic hairpin assembly; SWCNTs—single-walled carbon nanotubes; CNTs—carbon nanotubes; CoFe2O4 MNPs—CoFe_2_O_4_ magnetic nanoparticles; SWV—square wave voltammetry; GNF@Pt—gold nanoflower/platinum; AuNRs—gold nanorods; CeO_2_—cerium dioxide; MWCNTs-COOH—carboxyl multi-walled carbon nanotubes; MoS_2_—molybdenum disulfide; rGO—reduced graphene oxide; CuCo_2_O_4_—nanoporous copper-cobalt oxide hollow spheres; GONRs—graphene oxide nanoribbons; Ag-PEI NPs—polyethyleneimine-silver nanoparticles; CV—cyclic voltammetry; Cu-NMOF—copper-based metal organic framework; PtNPs—platinum nanoparticles; GQDs—graphene quantum dots; ERGO—electrochemically-reduced graphene oxide; AuNWs—gold nanowires; BPNSs—black phosphorus nanosheets; C_60_—fullerene; SSWV—stripping square wave voltammetry; pNHCSs—nitrogen-doped hollow carbon nanospheres with large pores.
